# The Metropolitan Hospital Sunday Fund

**Published:** 1913-02-15

**Authors:** 


### THE METROPOLITAN HOSPITAL SUNDAY FUND.

The leading article we published under this title in our issue of December 28 last has borne excellent fruit. A special meeting of the Council of this Fund was held on January 29 last, when it was unanimously decided to appoint a small committee, to which the laws of the constitution were referred, with instructions to bring up rules or bye-laws regulating the procedure which shall in future govern the proceedings at the meetings of the constituents of the Fund. These bye-laws, when approved by the Council, will be laid before a special meeting of the constituents to be held at the earliest possible date. At the same Council meeting it was unanimously resolved to summon a special meeting of the constituents at the Mansion House on February 10 to consider the resolutions of which notice had been given by the Rev. L. S. Lewis, of St. Mark's, Whitechapel, before the meeting of constituents last December, when they could not be dealt with for reasons already published. We give a full account of the meeting of constituents on the 10th instant, which filled the Egyptian Hall, and was most encouraging from the circumstance that it demonstrated the keen interest felt in the Sunday Fund by the clergy and ministers of all denominations throughout London and their congregations. It is unnecessary to dwell here upon the bearings of the resolutions submitted by Mr. Lewis, because their relevancy had been destroyed by the appointment by the Council of the Committee on Bye-laws just referred to. We may, however, point out that if the third of Mr. Lewis's resolutions, which deleted the words "in the month of December" from Law 1 of the constitution, had been adopted, it would have destroyed, as the laws of the constitution at present stand, the control over the awards at present vested in the constituents by placing in their hands the power to elect the Council of this Fund, the most important duty of which is each year to select and appoint a Committee of Distribution.

Mr. Lewis missed a great opportunity in not rising to the occasion by accepting the appointment of the Committee on Bye-laws and claiming that appointment as the fruit of his persistent efforts to effect this object, together with the better regulation of the proceedings at the annual meetings which must now result. Mr. Stephen Coleridge, with his usual astuteness, has hastened to attempt to take all the credit to himself, which his follower, Mr. Lewis, omitted to claim, by writing a characteristic letter to the *Morning Post*, which appeared on the 11th instant at the foot of the report of the proceedings at the Mansion House. The omission of Mr. Lewis and the action of Mr. Stephen Coleridge conclusively enforce the accuracy of Mr. Sydney Holland's contention that the interests of the Anti-Vivisectionist Hospital, rather than the interests of the Sunday Fund, have underlain much of the agitation which we hope was finally ended at the Mansion House on the 10th instant. Sir David Burnett, the present Lord Mayor, made an admirable Chairman—quick, just, impartial, and firm. He made a meeting, which in less skilled hands might have led to regrettable scenes, one which must be memorable in the history of the Sunday Fund as marking a notable departure, instinct with new life and hope for the hospitals and the Fund. The City may well be proud of Sir David Burnett and hospital managers, and the clergy and ministers of all denominations will gratefully recognise the debt of gratitude they owe to him as the President of the Metropolitan Hospital Sunday Fund.

